# ENDOGLIN Is Dispensable for Vasculogenesis, but Required for Vascular Endothelial Growth Factor-Induced Angiogenesis

**DOI:** 10.1371/journal.pone.0086273

**Published:** 2014-01-28

**Authors:** Zhen Liu, Franck Lebrin, Janita A. Maring, Sander van den Driesche, Stieneke van der Brink, Maarten van Dinther, Midory Thorikay, Sabrina Martin, Kazuki Kobayashi, Lukas J. A. C. Hawinkels, Laurens A. van Meeteren, Evangelia Pardali, Jeroen Korving, Michelle Letarte, Helen M. Arthur, Charles Theuer, Marie-José Goumans, Christine Mummery, Peter ten Dijke

**Affiliations:** 1 Department of Molecular Cell Biology, Cancer Genomics Centre, Centre for Biomedical Genetics, Leiden University Medical Center, Leiden, The Netherlands; 2 Hubrecht Institute, Utrecht, The Netherlands; 3 Department of Anatomy and Embryology, Leiden University Medical Center, Leiden, The Netherlands; 4 Center for Interdisciplinary Research in Biology (CIRB), CNRS UMR 7241/INSERM U1050, Collège de France, Paris, France; 5 Molecular Structure and Function Program, The Hospital of Sick Children, Department of Immunology and Heart and Stroke Richard Lewar Center of Excellence, University of Toronto, Toronto, Ontario, Canada; 6 Institute of Genetic Medicine, Newcastle University, International Centre for Life, Newcastle upon Tyne, United Kingdom; 7 Tracon Pharmaceuticals, San Diego, California, United States of America; University of Michigan, United States of America

## Abstract

ENDOGLIN (ENG) is a co-receptor for transforming growth factor-β (TGF-β) family members that is highly expressed in endothelial cells and has a critical function in the development of the vascular system. Mutations in *Eng* are associated with the vascular disease known as hereditary hemorrhagic telangiectasia type l. Using mouse embryonic stem cells we observed that angiogenic factors, including vascular endothelial growth factor (VEGF), induce vasculogenesis in embryoid bodies even when *Eng* deficient cells or cells depleted of *Eng* using shRNA are used. However, ENG is required for the stem cell-derived endothelial cells to organize effectively into tubular structures. Consistent with this finding, fetal metatarsals isolated from E17.5 *Eng* heterozygous mouse embryos showed reduced VEGF-induced vascular network formation. Moreover, shRNA-mediated depletion and pharmacological inhibition of ENG in human umbilical vein cells mitigated VEGF-induced angiogenesis. In summary, we demonstrate that ENG is required for efficient VEGF-induced angiogenesis.

## Introduction

During development of the embryo, blood vessels evolve *de novo* from hemangioblasts that differentiate into endothelial cells and form a primary vascular plexus. This process is defined as vasculogenesis [1]. Angiogenesis refers to the remodeling and maturation of this primitive vascular network into a branched vascular network [2]. Angiogenesis is a dynamic and carefully balanced process involving an activation phase associated with increased vascular permeability, basement membrane degradation, endothelial proliferation and migration, and a resolution phase accompanied by inhibition of endothelial cell proliferation and migration, in parallel with basement membrane reconstitution [3]. In the maturation phase the recruitment of pericytes and vascular smooth muscle cells is needed to maintain vessel stability and protect endothelial cells from apoptosis [4], [5].

Vascular endothelial growth factor (VEGF) plays a very prominent role in vasculogenesis and angiogenesis. VEGF represents a family of related cytokines, of which the VEGF-A isoform is a potent endothelial mitogen strongly induced by hypoxia [6]. Mice lacking one *Vegfa* allele die at embryonic day (E)8.5 as a result of vascular malformations [2], [7]. VEGF-A signaling occurs via the high affinity tyrosine kinase receptors VEGFR1 (FLT-1), and VEGFR2 (FLK-1) [8], [9]; VEGFR2 is the important endothelial VEGF receptor during angiogenesis. *Vegfr2* knockout mice die at E8.5 from impaired development of hematopoietic and endothelial cells [10] and closely resemble VEGF-A deficient embryos.

Endoglin (ENG or CD105) is a transmembrane glycoprotein essential for angiogenesis and vascular development, which is predominantly expressed in vascular endothelial cells [11]. Mice lacking *Eng* die at El0.5-E11.5 from angiogenic and cardiovascular defects. The early steps of vasculogenesis appear to be normal but the primary endothelial network fails to remodel into a mature circulatory system [12]–[14]. ENG functions as a co-receptor for transforming growth factor-β (TGF-β) family members, and interacts with their signaling serine/threonine kinase receptors [15], [16]. TGF-β relays its signal via Type I receptors (TβRI), also termed as activin receptor-like kinases (ALKs). TβRI acts downstream of type II receptors (TβRII) [17] and mediates the activation of intracellular SMAD effector transcription factors [18]. In endothelial cells, TGF-β can signal via two different TβRIs, ALK1 and ALK5 [3], [19]. Activation of ALK1 induces SMAD1 or −5 phosphorylation and mediates endothelial cell proliferation and migration, whereas ALK5 induces SMAD2 and −3 activation leading to vascular quiescence [3], [20]. ENG promotes ALK1/Smad1/5 signaling and inhibits ALK5/SMAD2/3 signaling [21]–[23]. ENG and ALK1 have also been shown to bind other TGF-β family members. Bone morphogenetic protein (BMP) 9, in particular, can bind directly and with high affinity to ENG and ALK1 [24], [25].

In humans, mutations in *Eng* lead to hereditary hemorrhagic telangiectasia type I (HHT1, also known as Rendu-Osler-Weber syndrome), while HHT2 is associated with mutations in the type I receptor, ALK1 [26], [27]. HHT is an inherited autosomal-dominant vascular disorder that affects the blood vessels of many organs. Characteristic symptoms include epistaxis (nosebleeds), skin and mucosal telangiectases associated with hemorrhage, as well as pulmonary, cerebral and hepatic arteriovenous malformations [28], [29].

During the differentiation of mouse embryonic stem cells (ESCs) *in vitro*, hematopoietic commitment within *Vegfr2* ^+^ precursor populations are characterized by *Eng* expression [30]. In particular, *Eng* is expressed during the progression from the *Vegfr2* ^+^ *Cd45* ^−^ to *Vegfr2* ^−^ *Cd45* ^+^ stage, marking the hemangioblast [31]. In *Eng* deficient ESCs, the number of hemangioblast precursors were reduced and myelopoiesis and definitive erythropoiesis were severely impaired, suggesting that the regulated expression of ENG functions to support lineage-specific hematopoietic development from VEGFR2^+^ expressing precursors [30], [31]. Additional studies with forced expression of ENG in ESCs and transcriptional profiling studies on ENG^+^ and VEGFR2^+^ expressing cells from E7.5 embryos further supported an important role for ENG in hematopoietic development [32], [33].

In the present study, we examined the role of ENG in vasculogenesis and angiogenesis using aggregates of ESCs known as embryoid bodies (EBs). We found that endothelial cell differentiation was not affected by a lack of ENG, but that VEGF-induced angiogenesis was severely impaired. The effects were dependent on the level of *Eng*: heterozygotes exhibited an intermediate phenotype, reminiscent of features in HHT1 patients. These results were validated and consolidated by shRNA-mediated *Eng* depletion and pharmacological ENG inhibition studies in endothelial cells. The impaired VEGF-induced endothelial cell sprouting in the absence of ENG might provide a suitable cell model to screen for drugs that can rescue this phenotype, which might lead to novel treatment modalities.

## Results

### Absence of *Eng* impairs organization of vascular structures in 15-day-old embryoid bodies

**Figure 1:**
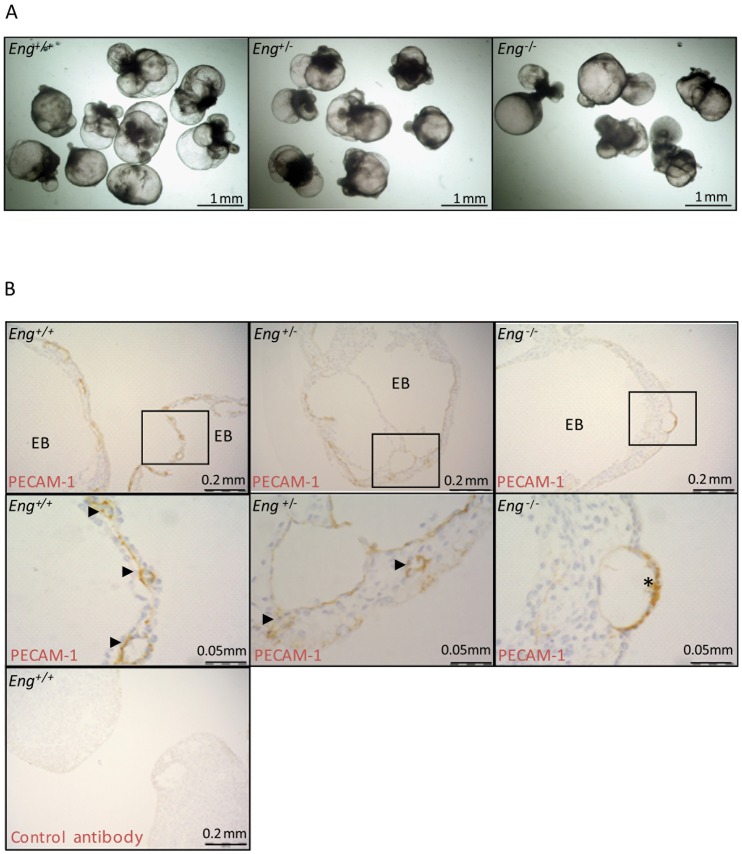
Impaired vasculature in *Eng* null-mutation ESC-derived 11-day-old EBs. (A) *Eng* ^+/−^ or *Eng* ^−/−^ ESC lines form EBs with no difference when compared to EBs derived from wild type ESCs (B) PECAM-1 whole mount immunohistochemistry of representative wild type, *Eng* ^+/−^, and *Eng* ^−/−^ ESC-derived 11-day-old EBs. Wild type ESC-derived EBs form a primitive vascular plexus. In contrast, *Eng* ^−/−^ ESC-derived EBs form irregular vascular structures with endothelial cell clusters. Light microscopy of serial plastic sections of wild type, *Eng* ^+/−^; and *Eng* ^−/−^ ESC-derived 11-day-old EBs stained as whole mount for PECAM-1. Black arrowhead indicates vessel like structures. Asterisk indicates endothelial cell clusters.

To elucidate the role of ENG in blood vessel morphogenesis we examined the effect of *Eng* gene dosage using the established assay of differentiation of ESCs into EBs [34]. When induced to differentiate, *Eng* ^+/−^ or *Eng* ^−/−^ ESC lines [13] were found to form EBs of similar size and compactness to those of wild type EBs (Fig. 1A). Next, the assembly of vascular structures was analyzed by platelet endothelial cell adhesion molecule (PECAM)-1 staining of sections of ESC-derived EBs with different *Eng* gene dosage (*Eng* ^+/+^, *Eng* ^+/−^ or *Eng* ^−/−^) obtained after 15 days of differentiation embedded in plastic and sectioned (Fig. lB). Morphology of the vasculature formed in wild type ESC-derived EBs was very similar to that of the yolk sac in wild type mouse embryos (Fig. 1B). Multiple blood islands, lined with a single layer of thin elongated endothelial cells, were found between the outer endoderm and the inner ectoderm layers (Fig. 1B), as reported previously by Wang *et al.* [35]. The number of blood islands in *Eng* ^−/−^ ESC-derived EBs appeared less numerous than in the wild type ESC-derived EBs and endothelial cells were found in clusters rather than in elongated single cell layers, confirming the defective formation of vessel-like structures in *Eng* ^−/−^ ESC-derived EBs (Fig. 1B). Vascular structures also developed in *Eng* ^+/−^ ESC-derived EBs, but their frequency and organization were markedly reduced compared to those in wild type ESC-derived EBs, indicating a dose dependent effect of *Eng* on vascular organization (Fig. 1B).

### ENG does not affect endothelial cell differentiation

**Figure 2:**
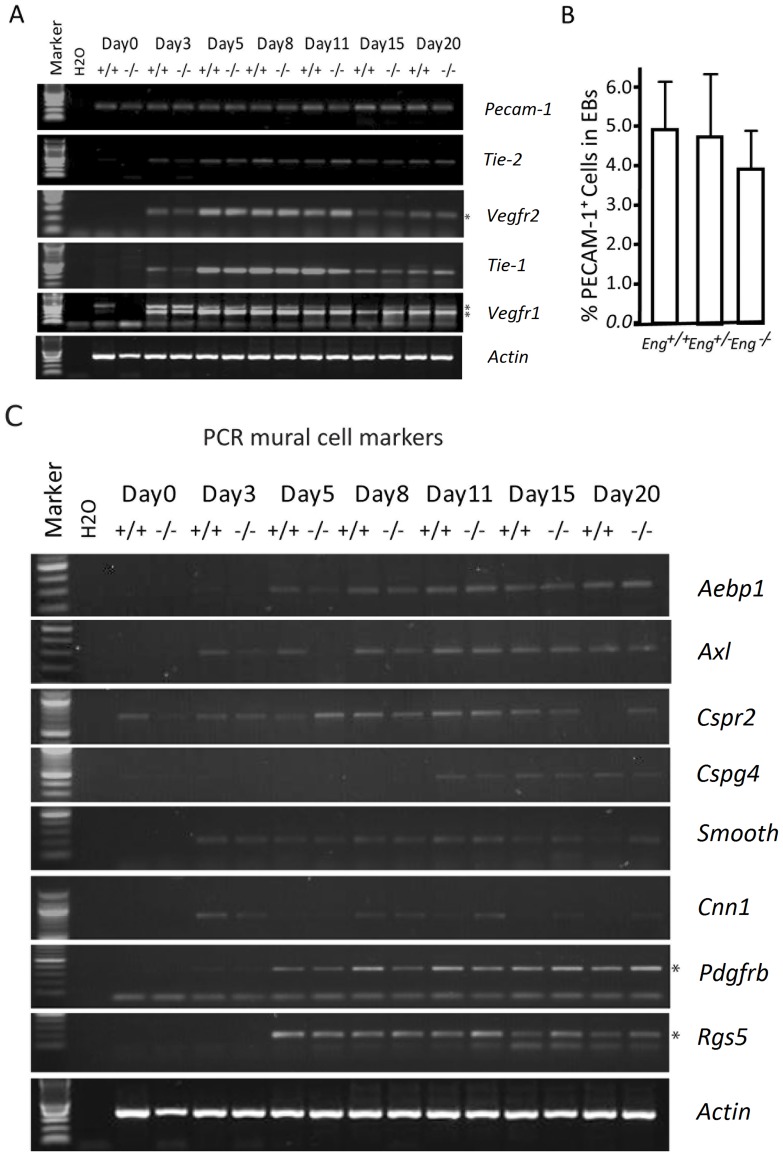
Expression of endothelial-specific markers during vascular development in EBs. (A) RT-PCR analysis of endothelial cell markers was performed on ESC and EBs cultured for the indicated number of days. Abbreviations: PECAM, platelet endothelial cell adhesion molecule; tie, tyrosine kinase with immunoglobulin-like loop and epidermal growth factor homology domain; VEGF, vascular endothelial growth factor receptor (B) The number of PECAM-1 positive cells was quantified by FACS analysis of the cell suspension of wild type, *Eng* ^+/−^, and *Eng* ^−/−^ ESC-derived 11-day-old EBs. (C) RT-PCR analysis of pericyte-smooth muscle cell markers was performed on ESC and EBs cultured for the indicated number of days. PCR primers used in this Figure are available upon request. Abbreviations: *Aebp*, adipocyte enhancer binding protein; *Axl*, a receptor tyrosine kinase; *Smooth*; Smoothelin B; *Cspr*, cysteine- and glycine-rich protein; *Cspg*, chondroitin sulfate proteoglycan; *Cnn*, calpoin; *Pdgf,* platelet-derived growth factor, *Rgs*, regulator of G protein signaling.

Two processes are responsible for the formation of blood vessels during embryonic development: (i) vasculogenesis, the primary *in situ* differentiation of endothelial precursors from mesoderm, and their organization into a primary capillary plexus and (ii) angiogenesis, the formation of new vessels by a process of sprouting from pre-existing vessels [1], [36]. RT-PCR analysis of endothelial cell specific markers on ESC-derived EBs collected from days 0 to 20 were used to define the role of ENG during endothelial cell differentiation. Distinct gene expression patterns were induced as differentiation proceeded. *Vegfr1* was rapidly up-regulated at day 3 and *Vegfr2*, *Tie-1* and *Tie-2* more prominently at day 5 (Fig. 2A). The expression patterns of the different EC markers were similar in *Eng* ^−/−^ ESC-derived EBs. In addition, we determined the number of PECAM-1 positive cells in dissociated 11-day-old EBs by FACS analysis and found no differences between wild type, *Eng* ^+/−^ or *Eng* ^−/−^ ESC-derived EBs (Fig. 2B). Analysis of the expression of multiple pericyte-vascular smooth muscle markers by RT-PCR also did not reveal striking differences between ESC-derived EBs with different *Eng* gene dosage (Fig. 2C). Taken together, our results show that ENG is not required for endothelial and mural cell differentiation.

### Endothelial cell organization is disrupted in *Eng* ^−/−^ ESC-derived EBs plated on gelatin

**Figure 3:**
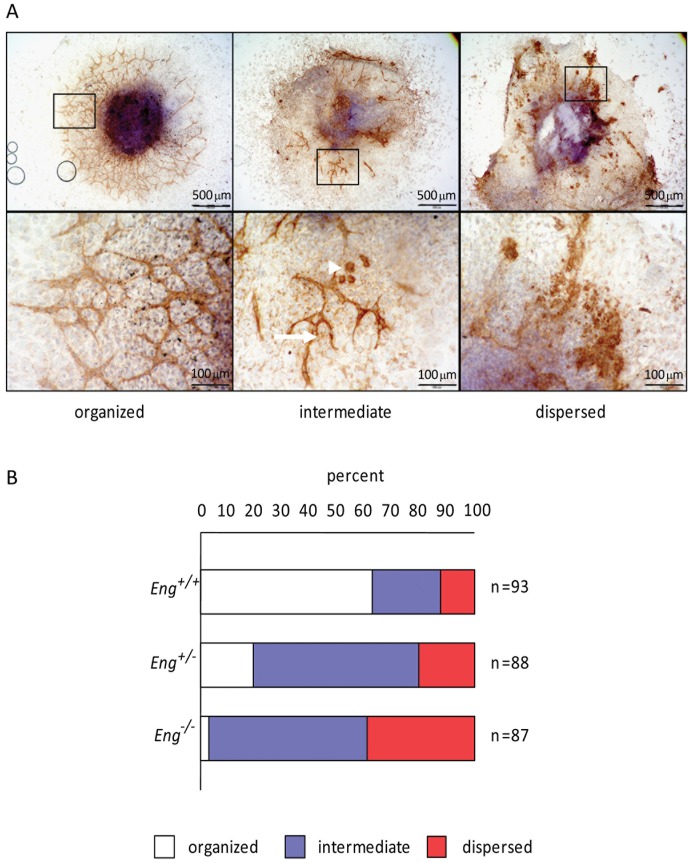
*Eng* ^−/−^ ESC-derived EBs plated on gelatin coated plates lack organized vessel structures. (A) PECAM-1 immunohistochemical staining of ESC-derived 11-day-old EBs plated on gelatin for 4 days displayed “organized”, “intermediate” or “dispersed” phenotype. (B) Quantification of wild type, *Eng* ^+/−^, and *Eng* ^−/−^ ESC-derived 15-day-old EBs vascular phenotypes as they were defined in A.

EBs plated on a gelatin-coated substrate can develop branching vascular structures indicative of vascular morphogenesis [37]. Endothelial cells are initially aggregated in dense clusters but when plated, rapidly form thin branching tubes, in a process resembling angiogenesis. To determine the role of ENG in this process, we plated 11-day-old EBs derived from *Eng* ^+/+^, *Eng* ^+/−^ and *Eng* ^−/−^ ESCs and maintained them in culture for four additional days before staining them with an antibody to PECAM-1 and hematoxylin to reveal the vascular network. Three different phenotypes could be identified in the EBs: (i) those with an extensively branched vascular network without endothelial cell clusters categorized as “organized”, (ii) those forming some vessels and still containing endothelial cell clusters referred to as “intermediate”, (iii) those with endothelial cells clusters only; these were designated as “dispersed” (Fig. 3A). Of around 90 EBs scored in each case in two independent experiments, on average about 63% of *Eng* ^+/+^ EBs showed an organized phenotype, ∼26% an intermediate phenotype and only ∼11% a dispersed phenotype (Fig. 3B). By contrast, in the *Eng* ^−/−^ EBs, ∼39% lacked cord-like structures entirely and were classified as dispersed, whereas ∼59% had an intermediate phenotype. Furthermore, the length of the vessel sprouts that did form was greatly reduced compared to those of the *Eng* ^+/+^ EBs and vessels appeared often wider. Quantitative analysis also showed that an intermediate vascular phenotype predominated in the *Eng* ^+/−^ EBs with ∼20% dispersed and ∼60% intermediate phenotypes (Fig. 3B). When EBs were embedded into a collagen gel and allowed to form vascular sprouts in 3D, we observed a reduction in both number of sprouts and sprout length in the *Eng* ^−/−^ EBs (Fig. S1).

**Figure 4:**
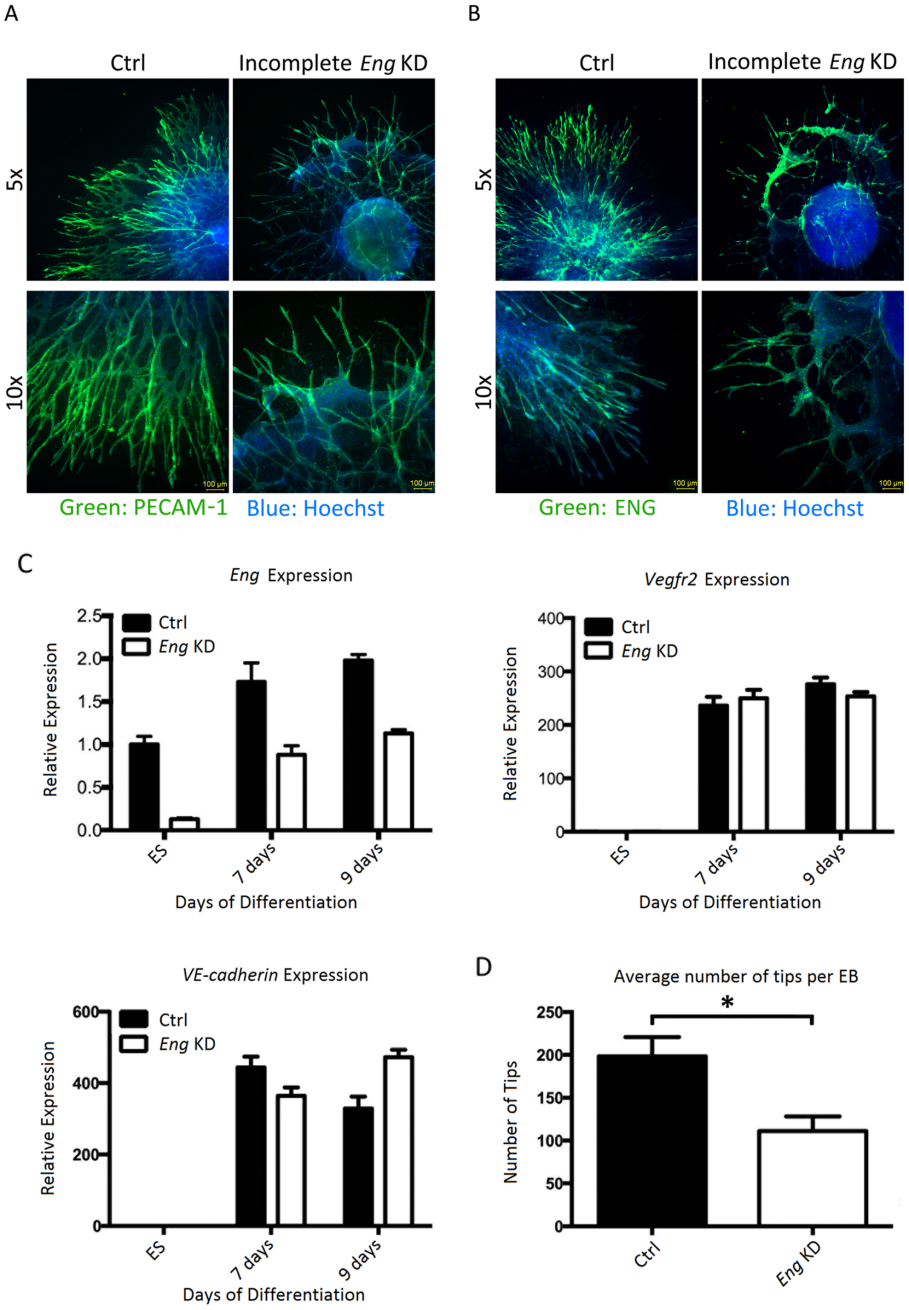
shRNA-mediated knock down of *Eng* inhibits VEGF-induced endothelial cell sprouting of EBs. ES cells were transduced with either scrambled or endoglin targeting shRNA. ES cells formed EBs during 4(30 ng/ml). Sprouting EBs were analyzed after 8 days of VEGF stimulation. A) Control EBs and EBs with incomplete endoglin knockdown stained for endothelial marker PECAM-1 (green) and DAPI (blue). Control EBs have large and many outgrowing sprouts of endothelial cells, which form extensive networks. EBs with incomplete *Eng* knockdown show less sprouts, which do not seem to form as extensive networks as control EBs. Sheets of cells that are mostly PECAM-1 negative have formed between the sprouts. B) Control EBs and EBs with incomplete *Eng* knockdown stained for ENG (green) and nuclear marker hoechst (blue). ENG is present in the entire sprout in the control EBs, with the highest expression towards the tip of the sprout. In the EBs with incomplete knockdown showed expression of ENG mainly in the tips of the outgrowing sprouts. The cellular sheets hardly had ENG expression. C) qPCR analysis of *Eng*, *PECAM-1* and *VE-cadherin* expression during differentiation of the control and *Eng* knockdown ES cells. Endoglin expression is reduced by approximately 85% in the ES cells, but during differentiation, at day 7 and 9, expression is restored to half of the normal levels. Expression of *Vegfr2* and *VE-cadherin* did not significantly differ between control and endoglin knockdown EBs at the ES cell state or at day 7 and 9 of differentiation. D) Analysis of number of tips per EB. Endoglin knockdown EBs exhibit significantly less sprouts than the control EBs (p<0.01).

To validate the data obtained with the *Eng* ^−/−^ ES cell line we depleted *Eng* by shRNAs targeting *Eng* in ESCs. Essentially we obtained the same results as for ESCs in which gene dosage was reduced (Fig. 4). Partial knock down of *Eng* in ESC in differentiated EBs (Fig. 4B, 4C) interfered with efficient VEGF-induced sprouting (Fig. 4D), whereas expression of endothelial markers *Vegfr2* and *VE-Cadherin* mRNA was not significantly affected (Fig. 4C).

### VEGF-induced angiogenesis is reduced in fetal metatarsals from *Eng^+/^* ^−^ mice

**Figure 5:**
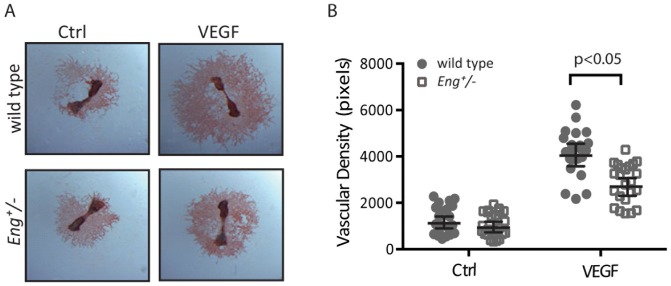
VEGF-induced angiogenesis is impaired in *Eng* ^+/^^−^ fetal metatarsal bones. Metatarsals of 17-day-old mouse fetuses were prepared from wild type and *Eng* ^+/−^ mice, transferred to cell-culture plates, allowed to adhere, and then stimulated with VEGF (50 ng/ml). (A) Cultures were fixed and vessel-like structures were visualized by anti-PECAM-1 staining. Six bones were stimulated per experimental group and one representative picture of each group is shown. (B) VEGF addition stimulated the formation of vessel-like structures. No significant difference in the baseline vascular network formation was observed between wild type and *Eng* ^+/−^ metatarsals. The induction of the vascular network of wild type metatarsals is significantly stronger than the network of *Eng* ^+/−^ metatarsals. *P*≤0.05.

In the studies above, VEGF was provided as the angiogenic stimulus. VEGF is a potent mitogen for endothelial cells and elevated ENG expression has been associated with activated endothelial cells in tumor stroma [38]. To investigate a possible interplay between VEGF and ENG in angiogenesis, we compared the VEGF-induced angiogenic response in fetal mouse metatarsals derived from wild type and *Eng* ^+/−^ mice. After adherence of the fetal bones to the culture dish, fibroblast-like cells migrate from the bones to form a monolayer, on which a tubular network of endothelial cells is formed [38], [39]. Staining of this endothelial cell network with an antibody to PECAM-1 showed that the VEGF-induced angiogenic responses, as measured by the number and the length of capillary sprouts were significantly reduced in the Eng^+/−^ metatarsals (Fig. 5). This result suggests that ENG is required for efficient VEGF-induced angiogenesis.

### Inhibition of ENG expression or function mitigates VEGF-induced sprouting of HUVECs

**Figure 6:**
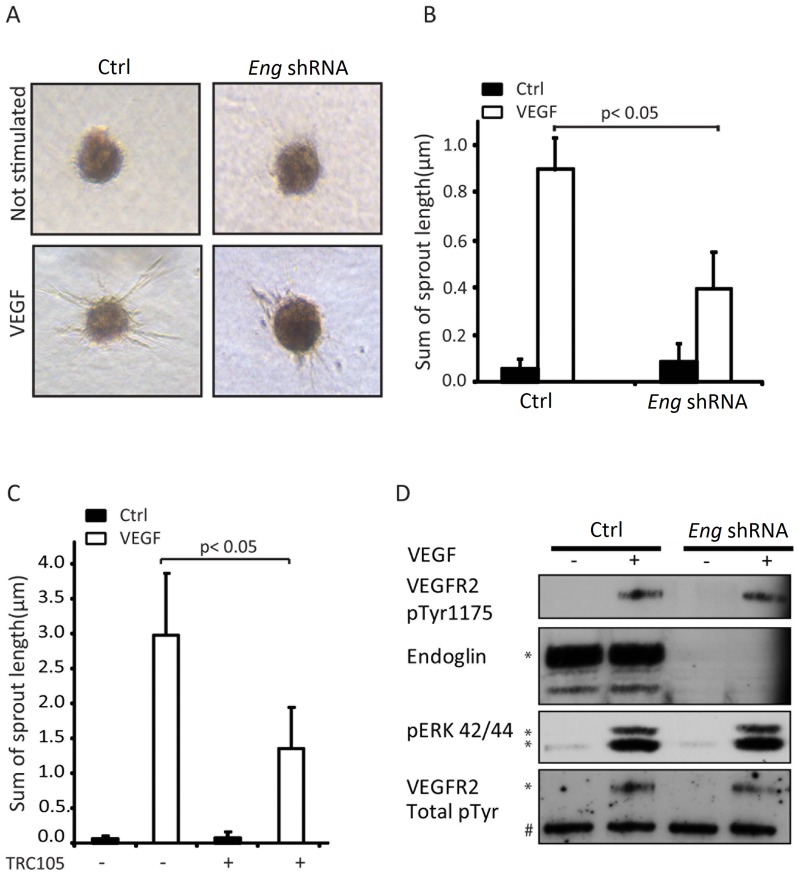
*Eng* deficiency inhibits VEGF-induced sprouting of HUVEC spheroids. (A) Effect of shRNA-mediated depletion of *Eng* on VEGF-induced endothelial cell sprouting. HUVECs were transduced with lentivirus expressing endoglin shRNA overnight. HUVEC spheroids with deficient endoglin expression were embedded in collagen and stimulated with VEGF (50 ng/ml). A representative experiment is shown. (B) Quantitation of effects seen in (A). (C) Effect of TRC105 ENG antibody on VEGF-induced endothelial cell sprouting. HUVEC spheroids were embedded in collagen and stimulated with VEGF (50 ng/ml), TRC105 (10 µg/ml), or both overnight. As control antibody for experiments using TRC105, the Fc domain (MOPC-21) from Bio Express, West Lebanon, NH, was used. Pictures were taken by phase-contrast microscopy. Quantitative analysis of the mean total sprout length was performed on 10 spheroids per experimental group. *P*≤0.05. (D) VEGF-induced VEGFR2 phosphorylation at site 1175 and extracellular regulated kinase (ERK) mitogen activated protein (MAP) kinase phosphorylation was examined in shRNA-mediated *Eng* knockdown cells. Two bands were detected with the phosphor ERK MAPK antibodies with a molecular weight of 44 and 42 kDa; they represent ERK1 and ERK2 isoforms, respectively. Number sign (#) represents a background band, indicating the even loading for the experiment. Asterisks indicate the protein bands with expected size.

To elucidate the role of ENG in VEGF-induced endothelial sprouting, we used a 3D-endothelial cell spheroid-sprouting assay, an established model for studying early *in vitro* angiogenic responses [40]. Non-stimulated spheroids of human umbilical vein endothelial cells (HUVECs) in collagen remain quiescent, and mimic the quiescent endothelial cells in the vessel wall. When stimulated with VEGF, tube-like protrusions emerge from the HUVEC spheroid within one day. We observed that shRNA-mediated depletion of *Eng* in HUVECs significantly reduced the VEGF-induced response in this assay (Fig. 6A, B). In addition, treatment of HUVECs with the ENG neutralizing antibody TRC105 also mitigated this response (Fig. 6C), confirming that inhibition of ENG function attenuates the VEGF-induced angiogenic response.

### Direct VEGF-induced signaling is not affected in *Eng*-deficient cells

The data above suggest an involvement of ENG in the VEGF signaling pathway or crosstalk between ENG and the VEGF pathway. We therefore further investigated the effect of ENG on VEGF signaling. The first event in the VEGF signaling cascade is binding of VEGF to its receptor, VEGFR2. However, specific depletion of *Eng* in HUVECs using shRNA did not affect VEGF binding to VEGFR2 as measured by affinity crosslinking with radiolabeled VEGF (data not shown). After VEGF-VEGFR2 interaction, VEGFR2 autophosphorylates itself at amino acid 1175, and thereafter initiates activation of the ERK kinase pathways. However, analysis of VEGF-induced VEGFR2, phospho-ERK pathways did not reveal any significant changes upon *Eng* knock down (Fig. 6D). These data indicate that ENG deficiency does not affect VEGF-induced ERK signaling directly.

## Discussion

In the present study, we examined the role of ENG in vasculogenesis and angiogenesis using aggregates of mouse ESCs known as EBs that were challenged with angiogenic supporting factors, including VEGF. Under appropriate conditions, both vasculogenesis and angiogenesis take place in EBs [41]–[45]. We compared EBs from wild type mouse ESCs with those from mouse ESCs with heterozygous or homozygous deletions in *Eng* (*Eng* ^+/−^ and *Eng* ^−/−^, respectively). We found that the endothelial cell differentiation program in ESC-derived EBs is not affected by homozygous deletion of *Eng.* However, homozygous mutant endothelial cells were severely inhibited in their ability to form organized vascular structures either following plating of EBs on gelatin in 2D or in 3D collagen gels, supporting evidence for an essential role of ENG in VEGF-mediated angiogenesis. This is consistent with reports by Bourdeau *et al.* [13], Li *et al*. [14] and Arthur *et al*. [12], and more recently by Park *et al*. [46]. However these data are different from earlier reports on the *Eng* ^−/−^ ESCs claiming no effect on endothelial cell organization in differentiating embryoid bodies. However, different methods were used, which might have contributed to the different outcomes [47]. To validate the defect in sprouting of *Eng* ^−/−^ ESC lines compared with control *Eng* ^+/+^ ESC, we depleted *Eng* by shRNA. Essentially we were able to confirm the results obtained using the knock out cells in that they are also defective in VEGF-induced endothelial cell sprouting, albeit not as dramatically as knock out cells. shRNA-mediated depletion has the advantage of looking at the effects of *Eng* depletion at an early stage, before any long term adaptation responses occur. Thus, we conclude that ENG is responsible for the lack of VEGF-induced endothelial vascular organization.

We observed interdependence for ENG in VEGF-induced angiogenic responses. Genetic depletion of *Eng* from endothelial cells and pharmacological inhibition using TRC105 ENG antibody severely affected VEGF-induced endothelial cell sprouting. These results are in line with previous studies, which demonstrated that ENG is essential for normal growth, migration and cord formation of endothelial cells [21], [48], [49]. In addition, our results are consistent with a recent report that showed that TRC105 inhibited VEGF and FGF-induced HUVEC endothelial tube formation when co-cultured with dermal fibroblasts [50]. Moreover, soluble ENG has been shown to inhibit tumor angiogenesis [51], [52], and elevated placental expression of ENG results in high serum levels of soluble ENG that contribute to vascular dysfunction in pre-eclampsia [53].

Remarkably, mouse embryonic endothelial cells (MEECs) isolated from *Eng* ^−/−^ embryos have been described as exhibiting enhanced proliferation [54]. The basis for the differences between our findings here and these studies is not clear. One explanation may be adaptive mechanisms that take place in endothelial cells in order to compensate for reduced ENG expression *in vivo* [21], [54]–[56].

Analysis of yolk sac vasculature in *Eng* mutant mice has shown previously that vascular smooth muscle cells are sparse in the vicinity of vessels lacking endoglin and it was striking that immunodetectable TGF-βl was reduced in the smooth muscle cells although TGF-βl mRNA levels in the adjacent endothelial cells were unaffected [55]. The impaired ability of endothelial cells to secrete or activate TGF-β1 was believed to explain the lack of phosphorylated Smad2 in the adjacent mesothelium and the subsequent failure of these cells to differentiate into vascular smooth muscle cells. In the EB vasculogenesis assay used here, vascular smooth muscle cells did form and organize to some extent, albeit abnormally, in the absence of *Eng* in contrast to the observations *in vivo*. However, the culture conditions used included the use of fetal bovine serum as a medium supplement, which could provide active TGF-β and facilitate partial rescue. Since the EB vasculogenesis assay closely models aspects of vascular development and includes both the differentiation and organizational aspects of EC and vascular smooth muscle cell components, it is potentially useful in screening anti- or pro-angiogenic drugs as well as in understanding the underlying molecular mechanisms.

In conclusion, our results provide insights into the molecular mechanisms that underlie vascular defects reminiscent of those in HHT1 patients and opens new avenues for inhibition of VEGF signaling by interfering with ENG function.

## Materials and Methods

### Cell culture

#### HUVECs

Human umbilical vein endothelial cells (HUVECs) cells were cultured in Medium 199 with Earle's salt and L-glutamine (Gibco), 10% FCS, heparin (LEO pharma), bovine pituitary extract (Gibco) and penicillin/streptomycin (PS) on plates coated with 1% gelatin, at 37°C and 5% CO_2_. HUVECs were used up to passage 4. Experiments were confirmed with HUVECs from different donors.

HUVECs were isolated from umbilical cords. The LUMC has the policy that umbilical cords are considered as “rest material” and collection can be performed without permission of the ethical committee, provided that the donor of the umbilical cord has signed a written consent and that collection and processing of the umbilical cord is performed anonymously. At the LUMC obstetric unit, where the umbilical cords were collected, the donor of the umbilical cord was asked to sign a formal waiver. The written consents are archived at the department of Obstetrics at LUMC and collection of the umbilical cord was performed anonymously.

#### Embryonic stem cell lines and culture

Two independent R1 ESC lines were used as controls. *Eng* ^+/−^ mouse embryonic stem cells (ESCs) were generated by gene targeting of the parental wild-type 129/Ola-derived E14 ES cell lines, deleting 609 base pairs (bp), including *Eng* exon 1 and its initiation codon and leaving the *Eng* promoter intact [13]. *Eng* ^−/−^ ESCs were derived *in vitro* from *Eng* **^+/^** ^−^ ESCs by selection with high concentrations of G418 [30]. Genomic DNA was isolated from ESC lines using standard techniques [57]. Primers *MEFI* and *MERl* amplify normal Exon 1 (300 bp) and primers *MEFR1* and *MEZR* amplify the recombinant product (476 bp), as previously described [13]. ESC lines were cultured in the presence of mouse embryonic fibroblasts (MEFs) in DMEM, supplemented with 20% heat-inactivated fetal bovine serum (FBS), 0.1 mM [3-Mercaptoethanol, lx non-essential amino acids and 1000 U/ml recombinant Leukemia Inhibitory Factor (LIF).

### Lentiviral transduction

HUVECs were infected with lentivirus encoding an shRNA sequence against human *Eng* (TRCN0000003273, TRCN0000003276) selected from the MISSION shRNA library (Sigma) and a third lentivirus encoding shRNA was generated in our lab [58]. R1-ES cells were infected with lentivirus encoding an shRNA targeting mouse *Eng* (TRCN0000094355, MISSION shRNA library Sigma). As a control, a non-targeting shRNA sequence (SHC002) (Sigma) or empty vector pRRL was used. Virus transduction was performed overnight, and the infected cells were selected using culture medium containing puromycin (1 μg/ml) for 48 h. The efficiency of *Eng* knockdown was verified by qPCR.

### *In vitro* differentiation of embryonic stem cell clones

Two different methods were used to differentiate ES cells *in vitro.*

*Method 1*: ESC lines were cultured in hanging drops to form EBs, as described previously [59]. Briey, 800 cells were cultured in 20 µl of DMEM, supplemented with 20% FBS, 25 ng/ml VEGF, 50 ng/ml bFGF-2, hanging from the lid of the culture dish for 5 days, which allows the formation of cell aggregates (EBs). This makes it possible to control the size of the EBs and circumvents paracrine stimulation between EBs, and therefore allows a very high degree of reproducibility. Subsequently, EBs were either (i) cultured in suspension on bacterial dishes coated with 1% agar for 11 or 15 days. EBs were then washed with PBS and fixed in methanol (MeOH)-dimethyl sulfoxide (DMSO) in a ratio of 4:1, overnight (o/n) at 4°C before staining; or (ii) 11-day old EBs were plated on gelatin coated coverslips for 4 days and then fixed in Zinc fixative o/n at 4°C before staining.

*Method 2*: Mixed Feeder-ES cell cultures were trypsinized and subsequently cultured for 45 minutes on gelatin-coated plates before the experiment in order to deplete the MEFs, which adhere faster to the plate. The ES cells were harvested and plated in suspension as hanging drops of 20 µl in complete ES-medium, containing 1200 cells/drop, for four days.

### Embryoid body maturation in 2D culture

Four-day old EBs obtained with method 2 were plated in gelatin-coated 6 well-plates with 15–20 EBs per well. EBs were cultured in ES medium without LIF and supplemented with 50 ng/ml hVEGF-165 (PeproTech, Rocky Hill, USA). After 7 or 9 days, EBs were washed with PBS and RNA was isolated for qPCR analysis.

### Embryoid body maturation in 3-D Collagen matrix

*Method 1*: All the ingredients of the collagen medium (DMEM, 20% FBS, 25 ng/ml VEGF, 50 ng/ml bFGF-2) with the exception of collagen were mixed and stored on ice before harvest of the EBs to avoid prior polymerization of the medium. Prior to use, rat tail type 1 collagen was added and mixed to a final concentration of 1.25 mg/ml. 11-day-o1d EBs were immediately incorporated into the collagen medium at a final concentration of 50 EBs/ml. 12 ml was poured into a 35 mm bacterial grade Petri dish and cultures incubated for 3 days at 37°C in a 5% CO_2_ atmosphere [30], [42]. For further analysis of sprouting vessels, the 35 mm gel dish was inverted over a 50 mm × 75 mm glass slide. The collagen gel was gently laid out on the slide and excess liquid around the gel removed by pipetting with a dispenser. The gel was then dehydrated using nylon linen and absorbent filter cards. The slide was air-dried for 12 hours and incubated in zinc fixative o/n at 4°C before staining as previously described [42]. The EBs were stained for PECAM-1 (Clone MEC13.3, BD Biosciences).

*Method 2*: Collagen solution was made as following: Purecol (Advanced Biomatrix, San Diego, USA) with 34.65% HAM's F12 (Gibco), 6.25% NaOH (0.1M), 6.25% 10x F12, 1.25% 4-(2-hydroxyethyl)-1-piperazineethanesulfonic acid (HEPES) (1M), 0.975% Sodium bicarbonate 7.5% and 0.625% Glutamax. Four-day old EBs were suspended in 350 µl collagen and transferred to a 24-well collagen pre-coated plate (one EB per well). EBs were cultured in complete ESC-medium without LIF and supplemented with 30 ng/ml hVEGF-165. The EBs were cultured for eight days in collagen and the medium was changed every four days. Afterwards, EBs in collagen were washed with phosphate buffered saline (PBS) and fixed with 4% paraformaldehyde in PBS for 30 min at room temperature.

### RNA isolation and quantitative PCR

Total DNA-free cellular RNA was extracted with Trizol reagents, according to manufacturer's protocol (Invitrogen). Samples were DNase I treated to eliminate genomic DNA and 1 µg RNA was reversed transcribed as described [60]. All PCR analyses of the endothelial cell and SMC specific markers were as previously described [61]. RNA from ESCs and EBs from method 2 was isolated with the NucleoSpin RNA II kit according to manufacturer's protocol (Bioké, Leiden, Netherlands). qPCR was performed with SYBRGreen reagent (Roche) for *Eng*, *Vegfr2* and *VE-cadherin*. See Table 1 for sequences. The ΔΔCt method was applied for the expression profiling. Gene expression is normalized to house-keeping gene GAPDH and wild type ES cells as the reference sample.

**Table 1:** qPCR primer sequence.

Target	Forward primer	Reverse primer
*Eng*	GGTCATGACTCTGGCACTCA	AGGCGCTACTCAGGACAAGA
*Vegfr2*	ACCAAGGCGACTATGTTTGC	GGGCAAGTCACTTCAATGGT
*VE-cadherin*	ATTGAGACAGACCCCAAACG	TGTTTTTGCCTGAAGTGCTG
*GAPDH*	AACTTTGGCATTGTGGAAG	ACACATTGGGGGTAGGAACA

### Immunofluorescence staining

For cryosections, ESC-derived l5-day-old EBs were processed as previously described [62] and subsequently sectioned at 7 µm before acetone fixation for 10 minutes at 4°C, followed by 30 minutes air drying at RT. Next, slides were permeabilized for 5 minutes with 0.2% Triton X-100 in PBS, followed by blocking with 2% BSA in PBS at RT for l hour. The slides were then incubated with rat anti-mouse PECAM-1 (Clone MEC14.7, Santa Cruz) o/n at 4°C. The slides were then washed four times in PBS and incubated for 1 hour with goat anti-rat Cy3 (Jackson ImmunoResearch Laboratories) at RT. The slides were then washed four times in PBS and mounted in Mowiol before confocal laser microscope analysis. Slides containing EBs cultured in 3D-collagen gel and zinc fixed were permeabilized for 15 minutes with 0.2% Triton X-100 in PBS, followed by blocking with TNB blocking solution for 1 hour at RT, as described above. EBs were stained with rat anti-mouse PECAM-1 (Clone MEC13.3, BD Biosciences). The slides were then washed in TBS and incubated 1 hour with donkey anti-rat FITC (Jackson ImmunoResearch Laboratories) and goat anti-mouse (Jackson IrnmunoResearch Laboratories) secondary antibodies diluted in TNB. The slides were then washed four times in TBS and mounted in Mowiol before confocal laser microscope analysis. EBs matured in 3D according to *method 2* were excised from collagen. The EBs were blocked in blocking solution (Tris-Buffered Saline Tween (TBST) with 3% BSA) for 2 hours or overnight at room temperature. Staining with primary and secondary antibodies was done overnight. The EBs were stained with Hoechst to visualize the nuclei. The following antibodies were used: rat anti-mouse PECAM-1 (BD Pharmingen) and rat anti-mouse ENG (CD105 MJ7/18, BD Pharmingen). Secondary antibodies: donkey anti-rat Alexa 488 (Invitrogen) and goat anti-rabbit Alexa 594 (Invitrogen). EBs were stored at 4°C in PBS until analysis with fluorescence microscopy. Endothelial cell sprouting from the EBs was quantified by counting the number of sprouts per EB.

### Flow Cytometric analysis

To obtain single cell suspensions for FACS analysis, 15-day-old EBs were collected from agar coated-dishes and washed twice with PBS before being incubated for 30 minutes in a dissociation solution containing 0.2% collagenase B (Roche Diagnostics). EBs were gently ushed every 5 minutes using one ml tip. After centrifugation, the cell pellet was washed twice with 2% FBS in PBS and then incubated for one hour at 4°C with a FITC conjugated anti-mouse PECAM-1 before FACS analysis.

### Western blot analysis

HUVECs were seeded in six-well plates and allowed to grow to 90% confluence. Cells were washed with PBS and serum-starved for 5 hours. Cells were stimulated with VEGF 50 ng/ml for 5 minutes, washed with PBS and lysed in SDS sample buffer. Samples were boiled for 10 minutes and subjected to SDS-PAGE and western blotting. Phospho-VEGFRII, phospho-ERK antibodies were purchased from Cell signaling Technology. ENG was analyzed with an antiserum recognizing human ENG [63].

### 3D-culture spheroid assay

HUVECs (400 cells per spheroid) were suspended in Medium M199 containing Earle's salt and L-glutamine, 10% FBS, methylcellulose, heparin, bovine pituitary extract, PS and seeded in non-adherent round-bottom 96-well plates. After 24 hours, spheroids were embedded into collagen and stimulated with corresponding stimuli in the presence or absence of inhibitors or neutralizing antibodies for another 24 hours. As control antibody for experiments with ENG neutralizing antibody TRC105, the Fc domain (MOPC-21) from Bio Express, West Lebanon, NH, was used. EC sprouts were measured by Olympus Analysis software.

### *Ex vivo* fetal mouse metatarsal angiogenic assay

Metatarsals from 17-day-old mouse fetuses from *Eng* ^+/+^ and *Eng* ^+/−^ mice [12] were dissected as described previously [64]. Six metatarsals per experimental group were transferred to 24-wells tissue-culture plates containing α-MEM (Gibco), 10% FBS and penicillin/streptomycin (PS), and allowed to adhere for 4 days. Then, medium was replaced by fresh medium containing 50 ng/ml VEGF. Cultures were fixed 7 days after stimulation and vessel formation was visualized by anti-PECAM-1 staining [39]. Vascular density was quantified by automated image analysis with Image J. Animal experiments were approved by the Institutional Committee for Animal Welfare of the Leiden University Medical Center (LUMC) and were performed according to the regulatory quidelines.

### Statistics

All results are expressed as the mean ± s.d. Statistical differences were examined by two-tailed Student's t-test and P≤0.05 was considered to be statistically significant (in the figures, ^*^P≤0.05 and ^**^P≤0.01).

## Supporting Information

Figure S1 **Eng^−/−^ ESC derived EBs have impaired endothelial cell-derived vessel structures.** A) Bright field image and PECAM-1 staining of EBs from *Eng* ^+/+^ and *Eng* ^−/−^ ESCs. Both bright field image and the PECAM-1 staining show that the *Eng^−/−^* EB has less endothelial sprouts than the *Eng^+/+^* EB. B) Quantification of the number of sprouts per EB and length of the sprouts.
(TIF)
