# Supplementary figures and images for: ENDOGLIN Is Dispensable for Vasculogenesis, but Required for Vascular Endothelial Growth Factor-Induced Angiogenesis
Source: PLoS One. 2014 Jan 28;9(1):e86273. doi: 10.1371/journal.pone.0086273 (PMC3904881; doi:10.1371/journal.pone.0086273)

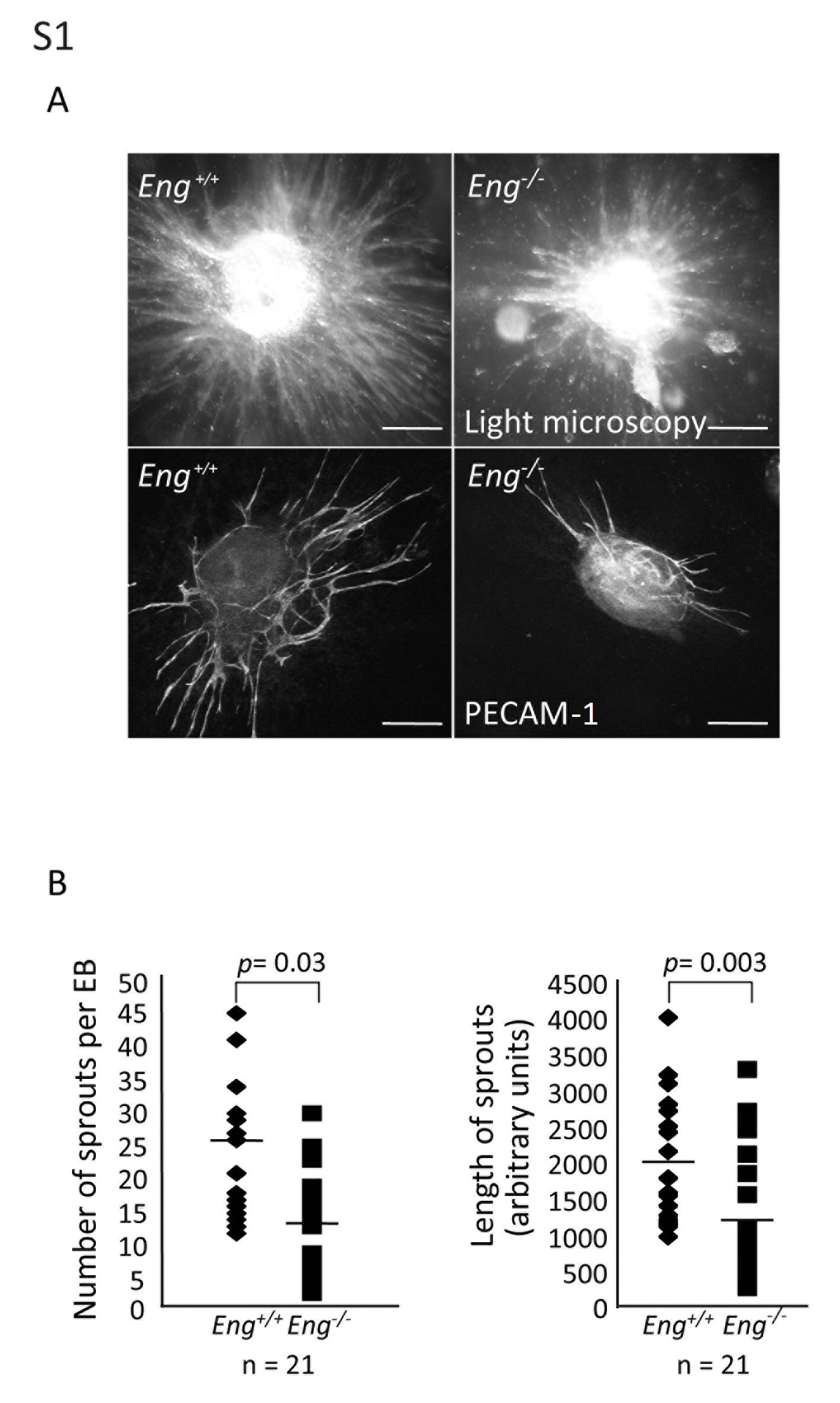

Supplement: Figure S1 — Eng−/− ESC derived EBs have impaired endothelial cell-derived vessel structures. A) Bright field image and PECAM-1 staining of EBs from Eng +/+ and Eng −/− ESCs. Both bright field image and the PECAM-1 staining show that the Eng−/− EB has less endothelial sprouts than the Eng+/+ EB. B) Quantification of the number of sprouts per EB and length of the sprouts. (TIF) [file pone.0086273.s001.tif]
